# Reduced Emotional Awareness and Distress Concealment: A Pathway to Loneliness for Young Men Seeking Mental Health Care

**DOI:** 10.3389/fpsyg.2021.679639

**Published:** 2021-06-21

**Authors:** David Kealy, Zac E. Seidler, Simon M. Rice, Daniel W. Cox, John L. Oliffe, John S. Ogrodniczuk, Dan Kim

**Affiliations:** ^1^Department of Psychiatry, University of British Columbia, Vancouver, BC, Canada; ^2^Orygen, Melbourne, VIC, Australia; ^3^Centre for Youth Mental Health, University of Melbourne, Melbourne, VIC, Australia; ^4^Counselling Psychology Program, University of British Columbia, Vancouver, BC, Canada; ^5^School of Nursing, University of British Columbia, Vancouver, BC, Canada; ^6^White Rock/South Surrey Mental Health & Substance Use Services, Fraser Health Authority, Surrey, BC, Canada

**Keywords:** loneliness, emotional awareness, distress concealment, disclosure, gender, age

## Abstract

**Background**: Loneliness, the painful affective state that reflects perceived deficits in social relationships, is a significant health issue requiring further understanding. Individual differences in awareness and disclosure of emotional concerns may contribute to loneliness, and may do so diversely according to gender and age. The present study examined a hypothesized mediation pathway from emotional awareness abilities to loneliness through distress concealment, with moderation by gender and age, in a sample of adults attending outpatient mental health services.

**Methods**: In a cross-sectional study design, 244 patients attending Canadian community mental health clinics completed study assessments at the commencement of care. Conditional process modeling examined interactions between gender and age and both emotional awareness and distress concealment in mediation models predicting loneliness.

**Results**: A significant three-way interaction between gender, age, and distress concealment was observed, along with significant conditional moderated mediation. The indirect effect of emotional awareness on loneliness through the mediating effect of distress concealment was significant for young- and mid-adulthood men, but not for women or older men.

**Limitations**: The study was limited by exclusive use of self-report assessment, and cross-sectional design precluding representation of causal sequencing over time.

**Conclusion**: Findings suggest the pathway to loneliness from reduced emotional awareness through distress concealment to be particularly salient for younger men. Thus, intervention targeting restricted awareness and disclosure of emotional concerns should be considered in helping young men to address the pain of loneliness.

## Introduction

Loneliness is a painful affective state attendant upon the subjective experience of inadequate social relationships. While loneliness may be evoked by an actual lack of relationships, it is possible to feel lonely surrounded by others or within a close relationship. Research has demonstrated loneliness to be associated with a multitude of mental and physical health problems (Cacioppo et al., 2015). Given recognition of loneliness as a significant public health issue, it is important to understand individual differences that contribute to susceptibility to loneliness. One such feature is emotional awareness. Limited awareness of emotional experience makes it harder to reveal emotional concerns to others (O’Loughlin et al., 2018), potentially reducing relationship quality (Cordova et al., 2005). The degree to which diminished emotional awareness, and the reduced disclosure of emotional distress, could impact loneliness may differ between men and women of different ages, given gender- and age-based social norms and roles regarding emotional experience and expression of vulnerability.

Emotional awareness is a trait-like ability to recognize and attend to emotional experiences, allowing for emotions to be incorporated in decision-making and interpersonal communication (Smith et al., 2018). Because social communication often involves emotional content or the evocation of affect, the capacity to attend to emotional experience may be an important prerequisite for satisfying interpersonal relationships. Research has shown reduced emotional awareness – and the related construct of alexithymia – to be associated with loneliness (Qualter et al., 2009; Eres et al., 2020). Limited emotional awareness may reduce one’s confidence in disclosing distress to others, which in turn may diminish the quality of one’s social connections. Disclosure of concerns can pave the way for others to provide understanding, validation, and support, thereby enhancing relationship quality (Cox et al., 2020). Given findings linking alexithymia with distress concealment (O’Loughlin et al., 2018), individuals with insufficient emotional awareness may limit their disclosures, jeopardizing connections with others and reducing avenues for social support. Indeed, previous research has shown distress concealment to be associated with loneliness (Wei et al., 2005; Cox et al., 2020). Thus, concealment of distress may serve as a mechanism between reduced emotional awareness and loneliness.

Although previous research points to limited emotional awareness and reduced distress disclosure as contributing to loneliness, few studies have investigated gender and age effects in relation to this pathway. Attentiveness to and communication of emotional experiences may reveal gender differences for outcomes, including loneliness (Nolen-Hoeksema, 2012), and loneliness may be shaped by stages of psychosocial development (von Soest et al., 2020). The consequences of disclosing or not disclosing emotional concerns may differ, for example, between young women and older men, on the basis of socialization and identity-related influences.

The present study investigated a hypothesized pathway from reduced emotional awareness to loneliness through the mediating effect of distress concealment, with the potential moderating effects of gender and age. This conditional moderated mediation model was examined among patients seeking mental health care. Due to its overlap with loneliness and near ubiquity among outpatients attending mental health services, general psychological distress was controlled for in testing the hypothesized model.

## Materials and Methods

### Participants and Procedures

Procedures and study reporting followed STROBE guidelines for observational cross-sectional studies. Participants were 244 adults attending three publicly-funded outpatient mental health clinics in the Greater Vancouver/Fraser Valley region of British Columbia, Canada. These clinics provide intervention for significant and impairing psychological distress such as depression, anxiety, interpersonal, and personality difficulties. Screening interviews by clinicians determine service eligibility, based on likely mental disorder with functional impairment; presentations of psychosis and primary substance use disorders are referred to alternate specialized care. Formal diagnoses are not provided since intervention – typically short-term individual or group psychotherapy – is guided by comprehensive case formulation. Participants in the study were consecutively admitted patients who provided informed consent following their initial screening to the clinic, and who completed study materials. Participants completed study measures upon commencement of care, while awaiting consultation. In order to mitigate potential bias, participants’ research data was not made available to clinicians. The study was approved by the affiliated research ethics boards, and data were collected between February 2017 and December 2018. Determination of sample size was guided by empirically-derived power considerations for mediation analyses (Fritz and MacKinnon, 2007). A total of 262 patients consented to participate, with 6.9% failing to complete measures for the present study, leaving a final sample of *N* = 244. Subsequent missing data represented less than 1% of values (among *n* = 10 participants); given minimal missing responses, these were imputed using the participant’s mean score for the remaining scale items.

### Measures

Due to concerns about study completion at patients’ entry to care in a busy clinic, we used brief psychometrically supported measures. The three-item Awareness subscale of the Difficulties in Emotion Regulation Scale-18 (DERS-18; Victor and Klonsky, 2016) was used to assess impaired emotional awareness, scored from 1 (*almost never*) to 5 (*almost always*). Higher total scores (*α* = 0.75) indicated lower emotional awareness. Distress concealment was assessed using three items from the Distress Disclosure Index (DDI; Kahn and Hessling, 2001) rated from 0 (*not at all like me*) to 4 (*extremely like me*). These items were selected from the 12-item DDI due to having the highest factor loadings of the items that explicitly asked about distress concealment. These items were summed (*α* = 0.87), with higher values representing greater distress concealment. Loneliness was assessed using a single item (“I feel completely alone”) rated on a five-point scale from 1 (*not at all characteristic of me*) to 5 (*extremely characteristic of me*). Meta-analytic evidence indicates that single-item measures of loneliness exhibit similar associations as measures such as the UCLA Loneliness Scale (Pinquart and Sörensen, 2001). General psychological distress was assessed using the Kessler Psychological Distress Scale (K6; Kessler et al., 2010), a six-item measure of depressive and anxiety symptoms experienced over the past month, scored from 0 (*none of the time*) to 4 (*all of the time*). Multiple studies support the K6 as a psychometrically sound measure of psychological distress; with higher scores indicating greater distress severity, and a score of 13 or higher indicating likely serious mental illness (Kessler et al., 2010; *α* = 0.89).

### Analyses

Descriptive statistics were used to characterize the sample and preliminary analyses examined gender and age effects on study variables. Conditional process modeling was performed using SPSS 25 and PROCESS 3.0 (Hayes, 2018). In our mediation model, emotional awareness difficulties was the independent variable, distress concealment was the mediator, and loneliness was the dependent variable – controlling for general distress in all paths. Gender and age were first- and second-stage moderators, allowing for three-way interaction. We used simple slopes to probe significant higher-order interactions at 16th, 50th, and 84th percentiles. Indirect effects of emotional awareness difficulties – indicating mediation by distress concealment – were evaluated using bootstrapped 95% CIs, resampled 10,000 times, and were considered significant if the CIs were absent zero. The conditioning effects of gender and age on indirect effects were evaluated using moderated mediation indices, also tested using bootstrapped 95% CIs.

## Results

More than half the sample, 63.9% (*n* = 156), were women, with men representing 35.7% (*n* = 87); one participant indicated non-binary gender. Participants’ average age was 40.38 years (*SD* = 14.81; range = 19–74 years). Regarding gender by age distribution, 14 men and 26 women were under 25 years old; 62 men and 101 women were between ages 25–56; and 11 men and 29 women were 57 years or older; the participant who identified as non-binary was 21 years old. The majority of patients, 39.7% (*n* = 97), reported employment; 18.8% (*n* = 46) were either retired, at-home parents, or students; 23.4% (*n* = 57) indicated being unable to work due to disability; and 17.6% (*n* = 43) were unemployed (*n* = 1 missing response). Less than half, 42.6% (*n* = 104), reported being in a committed relationship. Two-thirds of participants, 66% (*n* = 161), reported K6 scores ≥ 13, indicating likely serious mental illness, and most, 80.3% (*n* = 196), had previously sought psychotherapy. A minority of patients indicated an absence of, 13.9% (*n* = 34), or slight, 22.5% (*n* = 55) loneliness, while 22.5% (*n* = 55) indicated feeling moderately lonely. One-fifth, 20.9% (*n* = 51), reported feeling very lonely, and a further 20.1% (*n* = 49) endorsed extreme loneliness. Descriptive statistics consisted of emotional awareness difficulties, *M* = 8.389 (*SD* = 2.932); distress concealment, *M* = 7.018 (*SD* = 3.580); loneliness, *M* = 3.107 (*SD* = 1.338); and psychological distress, *M* = 14.776 (*SD* = 5.672). No bivariate gender or age associations were observed with any of the primary variables of interest.

**Figure 1:**
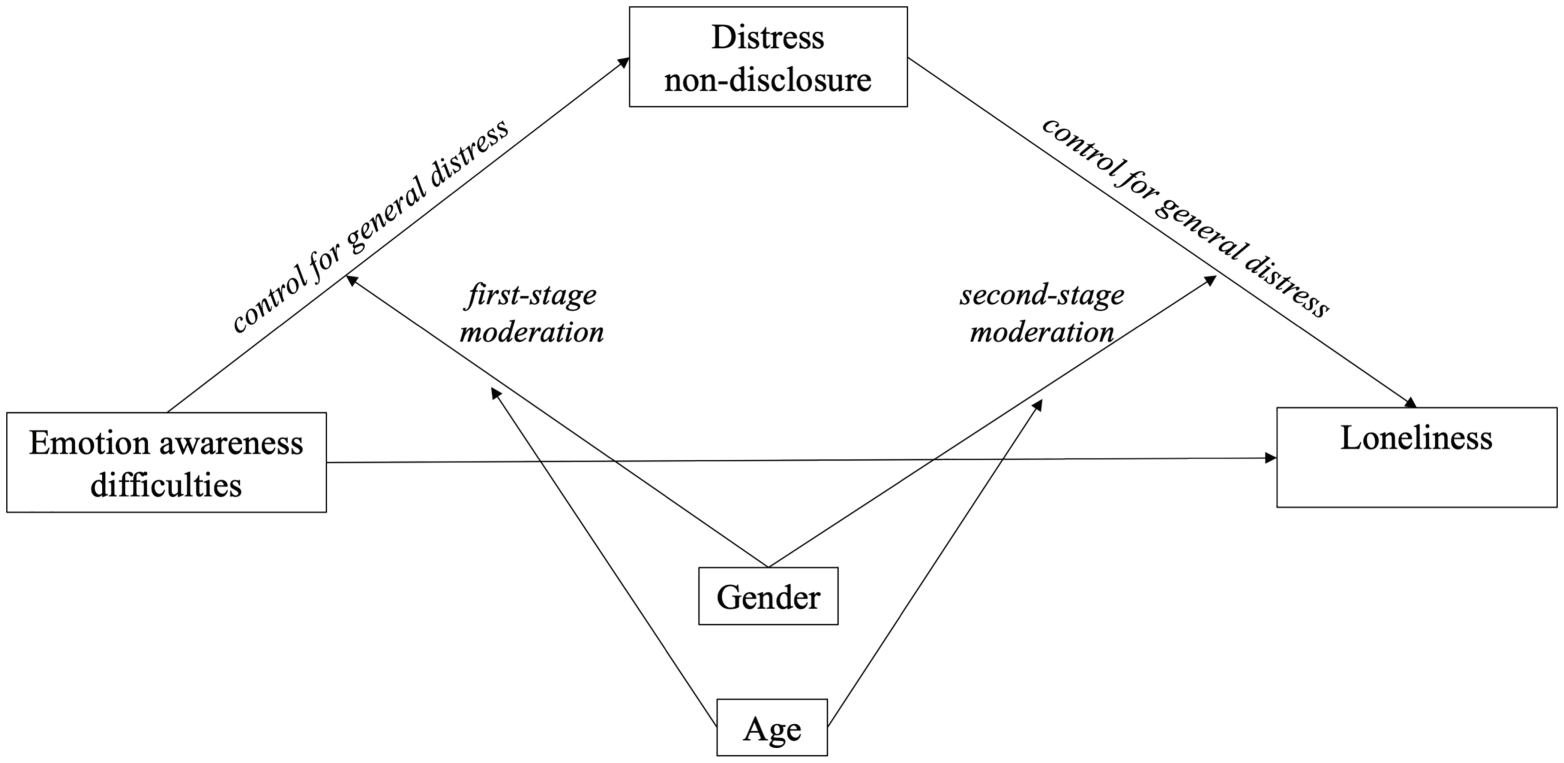
Illustration of conditional process analysis examining distress concealment as mediator of emotional awareness and loneliness.

Conditional process analysis examined interactions between gender (dummy coded male = 1), age, and emotional awareness (predicting distress concealment; *first-stage* moderation) and between gender, age, and distress concealment (predicting loneliness; *second-stage* moderation). This model (Figure 1) revealed a significant three-way interaction between gender, age, and distress concealment (i.e., in the path predicting loneliness), with significant conditional moderated indirect effects, *F*(10, 233) = 13.834, *MSE* = 1.172, *R*^2^ = 0.373, *p* < 0.001. Since no interaction at the first stage was evident, a parsimonious second-stage model was tested (i.e., with the non-significant interaction between gender, age, and emotional awareness removed), *F*(9, 234) = 14.471, *MSE* = 1.195, *R*^2^ = 0.358, *p* < 0.001, and reported in Table 1. Probing the significant interaction between distress concealment, gender, and age revealed that the positive association between distress concealment and loneliness was significant among men at the 50th percentile of age (38 years), *B* = 0.114, *SE* = 0.039, *t* = 2.956, *p* = 0.003, and at the 16th percentile (24 years), *B* = 0.189, *SE* = 0.060, *t* = 3.162, *p* = 0.002. Young men (age 24) high in distress concealment (84th percentile) tended toward the highest loneliness scores in the sample, while young men low in distress concealment (16th percentile) tended to evince the lowest loneliness scores. Regarding mediation, indices of conditional moderated mediation were significant (Table 1), indicating that gender and age moderated the indirect effects. Examination of 95% bootstrapped CIs revealed distress concealment as a significant mediator only for young- and mid-adulthood men (evident at age 24 and 38 years), with the mediation effect stronger among younger men.

**Table 1:** Regression coefficients (with SEs) examining second-stage conditional moderated mediation to loneliness.

	Outcome					
	Distress concealment	*t*	*p*	Loneliness	*t*	*p*
Impaired emotional awareness	0.417 (0.072)	5.826	**<0.001**	−0.000 (0.026)	−0.006	0.995
General psychological distress (covariate)	0.141 (0.037)	3.818	**<0.001**	0.130 (0.014)	9.455	**<0.001**
Distress concealment				−0.048 (0.072)	−0.671	0.503
Gender				−2.185 (1.075)	−2.033	**0.043**
Distress concealment × gender				0.366 (0.133)	2.745	**0.007**
Age				−0.010 (0.013)	−0.734	0.464
Distress concealment × age				0.002 (0.002)	1.073	0.284
Gender × age				0.045 (0.024)	1.905	0.058
Distress concealment × gender × age				−0.007 (0.003)	−2.399	**0.017**
						
**Indices of moderated mediation**	**Index**	**95% CI**				
Conditional moderated mediation, overall	−0.003 (0.001)	**−0.006**	**−0.001**			
Conditional moderated mediation, at 24 years	0.081 (0.028)	**0.032**	**0.139**			
Conditional moderated mediation, at 38 years	0.038 (0.017)	**0.007**	**0.075**			
Conditional moderated mediation, at 57 years	−0.019 (0.025)	−0.071	0.030			
Conditional moderated mediation, women	0.001 (0.001)	−0.001	0.002			
Conditional moderated mediation, men	−0.002 (0.001)	**−0.004**	**−0.001**			
						
	**Among men**			**Among women**		
**Indirect effects of emotional awareness difficulties**	**Estimate**	**95% CI**		**Estimate**	**95% CI**	
Through distress concealment, at 24 years	0.079 (0.023)	**0.039**	**0.127**	−0.002 (0.016)	−0.034	0.030
Through distress concealment, at 38 years	0.047 (0.015)	**0.021**	**0.080**	0.009 (0.011)	−0.013	0.033
Through distress concealment, at 57 years	0.005 (0.019)	−0.032	0.045	0.024 (0.018)	−0.007	0.063

CI = 95% bootstrapped CI sampled 10,000 times; significance indicated with boldface.

## Discussion

Findings from the present study indicated significant mediation from emotional awareness difficulties to loneliness – through distress concealment – that was conditional on age and gender. Mediation by distress concealment was only significant among young- and mid-adulthood men. This pathway to loneliness was not observed for older men or women of any age and was not attributable to the effects of general psychological distress. It is important to note that neither being male nor being younger was directly associated with loneliness. Rather, young male patients experienced more loneliness on the basis of their distress concealment, and linked indirectly with lower emotional awareness. By contrast, young men who tended to recognize their feelings and disclose their difficulties experienced less loneliness.

What is it about being young and male that intensifies the pathway to loneliness through reduced emotional awareness and disclosure? One possibility is that young men with underdeveloped emotional abilities may be more inclined to subscribe to traditional masculine norms that valorize independence and proscribe emotional disclosure (Seidler et al., 2016). These men may adopt a stereotypical version of masculine identity in lieu of one founded upon “knowing oneself,” but may ultimately experience its risk in terms of thwarted connectedness. Indeed, research has shown that compared to older men, younger men tend to endorse higher levels of externalizing symptoms – potentially reflecting distorted masculinity norms – which may interfere with social connection (Rice et al., 2019). By contrast, younger men with robust emotional awareness may be more aligned with pro-social values that bring them into greater contact with others (Oliffe et al., 2019). For these men, emotions and their disclosure may be welcome aspects of individual and interpersonal experience.

Practically, an implication of the present findings may indicate clinical attention to young men’s emotional awareness abilities and level of comfort disclosing personal concerns, potentially involving exploration of masculine identity (Seidler et al., 2018). Interventions targeting such features may help to reduce young men’s loneliness – a precursor to later psychosocial difficulties (von Soest et al., 2020) and potential risk factor for suicidality (Calati et al., 2019).

Limitations of the present study include the use of cross-sectional data and exclusively self-report and brief measures, including a single loneliness item that may not fully tap the multi-dimensions of loneliness. Information was also lacking regarding participants’ psychiatric diagnoses and presenting problems – precluding inferences about the potential effects of such variables. Nevertheless, the use of a clinical sample and the robust statistical approach to gender and age effects are strengths of this preliminary effort. Replication is needed in larger clinical samples, with more representation of younger and older individuals. The findings may stimulate further research into and clinical consideration of young men’s emotional awareness and disclosure difficulties as risks for loneliness.

## Data Availability Statement

The raw data supporting the conclusions of this article will be made available by the authors, without undue reservation.

## Ethics Statement

The studies involving human participants were reviewed and approved by Behavioral Research Ethics Board, University of British Columbia and the Research Ethics Board, Fraser Health Authority. Written informed consent for participation was not required for this study in accordance with the national legislation and the institutional requirements.

## Author Contributions

DKe: conceptualization, study design, data analysis, interpretation, and preparation of the article draft. DKe and DKi: data collection. ZS, SR, DC, JOl, JOg, and DKi: contribution to critical revision of the article draft. All authors contributed to the article and approved the submitted version.

### Conflict of Interest

The authors declare that the research was conducted in the absence of any commercial or financial relationships that could be construed as a potential conflict of interest.

The reviewer OL declared a past co-authorship with several of the authors DK, JO to the handling editor.
